# Newly identified invasion drivers define the tumor front in oral squamous cell carcinoma

**DOI:** 10.1038/s41420-026-03202-y

**Published:** 2026-06-20

**Authors:** Guillermo Flores, Slyn Uaroon, Juan A. R. Garay, Marion Vanneste, Jesse D. Riordan, Adam J. Dupuy, Kristina W. Thiel, Kaitriana E. Colling, Christopher S. Stipp, Li-Chun Lin, Bailey Hollis, Mariah R. Leidinger, Stephanie J. T. Chen, Lillian M. Graham, Kevin L. Knudtson, Michael S. Chimenti, Jason A. Ratcliff, Zain Mehdi, Madison C. McElliott, Eric R. Anderson, Michael D. Henry, Molly E. Heft Neal, J. Chad Brenner, Marisa R. Buchakjian

**Affiliations:** 1 Department of Otolaryngology-Head and Neck Surgery, University of Iowa, Iowa City, IA USA; 2 Holden Comprehensive Cancer Center, University of Iowa, Iowa City, IA USA; 3 Department of Molecular Physiology and Biophysics, University of Iowa, Iowa City, IA USA; 4 Department of Anatomy and Cell Biology, University of Iowa, Iowa City, IA USA; 5 Department of Obstetrics and Gynecology, University of Iowa, Iowa City, IA USA; 6 Department of Biology, University of Iowa, Iowa City, IA USA; 7 Iowa NeuroBank Core, Iowa Neuroscience Institute, Carver College of Medicine, Iowa City, IA USA; 8 University of Iowa Institute for Vision Research, Iowa City, IA USA; 9 Department of Pathology, University of Iowa Hospitals & Clinics, Iowa City, IA USA; 10 Iowa Institute of Human Genetics, Carver College of Medicine, University of Iowa, Iowa City, IA USA; 11 Carver College of Medicine, University of Iowa, Iowa City, IA USA; 12 Department of Otolaryngology-Head & Neck Surgery, University of Michigan, Ann Arbor, MI USA; 13 Rogel Cancer Center, University of Michigan, Ann Arbor, MI USA

**Keywords:** Oral cancer, Cancer genomics, Oncogenesis, Cancer models, Mechanisms of disease

## Abstract

Cells at the invasive front of oral squamous cell carcinoma (OSCC) occupy a partial epithelial-to-mesenchymal transition (p-EMT) state that drives invasion and therapeutic resistance yet remains poorly understood. To define the regulators of this metastable state, we developed an isogenic OSCC model that captures stable epithelial and p-EMT phenotypes and used it to perform a genome-wide CRISPR invasion screen. This unbiased strategy identified 17 modulators of invasion, most previously unrecognized in cancer and virtually unexplored in OSCC, that converge on cytoskeletal remodeling, adhesion, and epigenetic regulation. Within this network, EDIL3 emerged as a central driver of EMT, invasion, and chemoresistance in head and neck cancer, extending prior reports in other tumor types. Importantly, the invasion signature derived from our screen localized with striking specificity to the tumor invasive front of human OSCC, providing direct clinical validation. Together, these findings reveal a novel, targetable network of transient invasion drivers defining the OSCC tumor front.

## Introduction

Metastable cell states play a central role in cancer progression, driving invasion, metastasis, and treatment resistance [1–3]. At the leading edge of carcinomas, cells frequently adopt the dynamic program of partial epithelial-to-mesenchymal transition (p-EMT), which enables cancer cell infiltration and therapeutic evasion [4–7]. This phenomenon plays a particularly important role in oral squamous cell carcinoma (OSCC), the most common malignancy of the oral cavity, characterized by invasive behavior, high recurrence rates, and limited therapeutic options [8]. Several studies have demonstrated that subpopulations of OSCC cells expressing a p-EMT signature localize to the tumor–stromal interface and drive invasion, suggesting that epithelial and mesenchymal features may be modulated both spatially and temporally to produce aggressive transitionary cellular phenotypes [9–11]. However, these transient cell states remain difficult to capture and interrogate using conventional approaches. Large-scale genomic profiling has advanced our understanding of OSCC biology, identifying recurrent mutations in genes such as *TP53*, *CDKN2A*, and *FAT1* [12]. Yet, most alterations are loss-of-function events, yielding few clinically actionable targets, and such studies are not optimized to uncover causal regulators of complex processes like invasion, where spatial and temporal contexts are paramount. Furthermore, although single-cell technologies have significantly improved our understanding of p-EMT, these methods primarily provide descriptive snapshots rather than directly interrogate the molecular mechanisms by which invasive front cells drive disease by transiently modifying gene expression.

In this study, we establish a novel isogenic OSCC model that isolates the normally ephemeral p-EMT transcriptional signature. By comparing epithelial and p-EMT subpopulations, we delineate the transcriptional and epigenetic programs that distinguish this transient invasive state. We then leverage this powerful model to develop a new genome-wide invasion screen, enabling the unbiased identification of critical modulators that either promote or restrict basement membrane invasion. Most of these regulators have not been thoroughly studied in OSCC, likely due to the limitations of traditional genomic and transcriptomic approaches described above. Our model supports OSCC invasion being driven by a p-EMT cell state defined by extensive cytoskeletal remodeling and extracellular matrix reprogramming to create an invasion-permissive phenotype reinforced by epigenetically regulated signaling. Finally, we demonstrate the translational power of our model by showing that the pro-invasion signature derived from our screen localizes to the invasive front of OSCC tumors in situ, underscoring its clinical relevance.

## Results

### A stable p-EMT OSCC model is characterized by increased invasion and treatment resistance

**Fig. 1:**
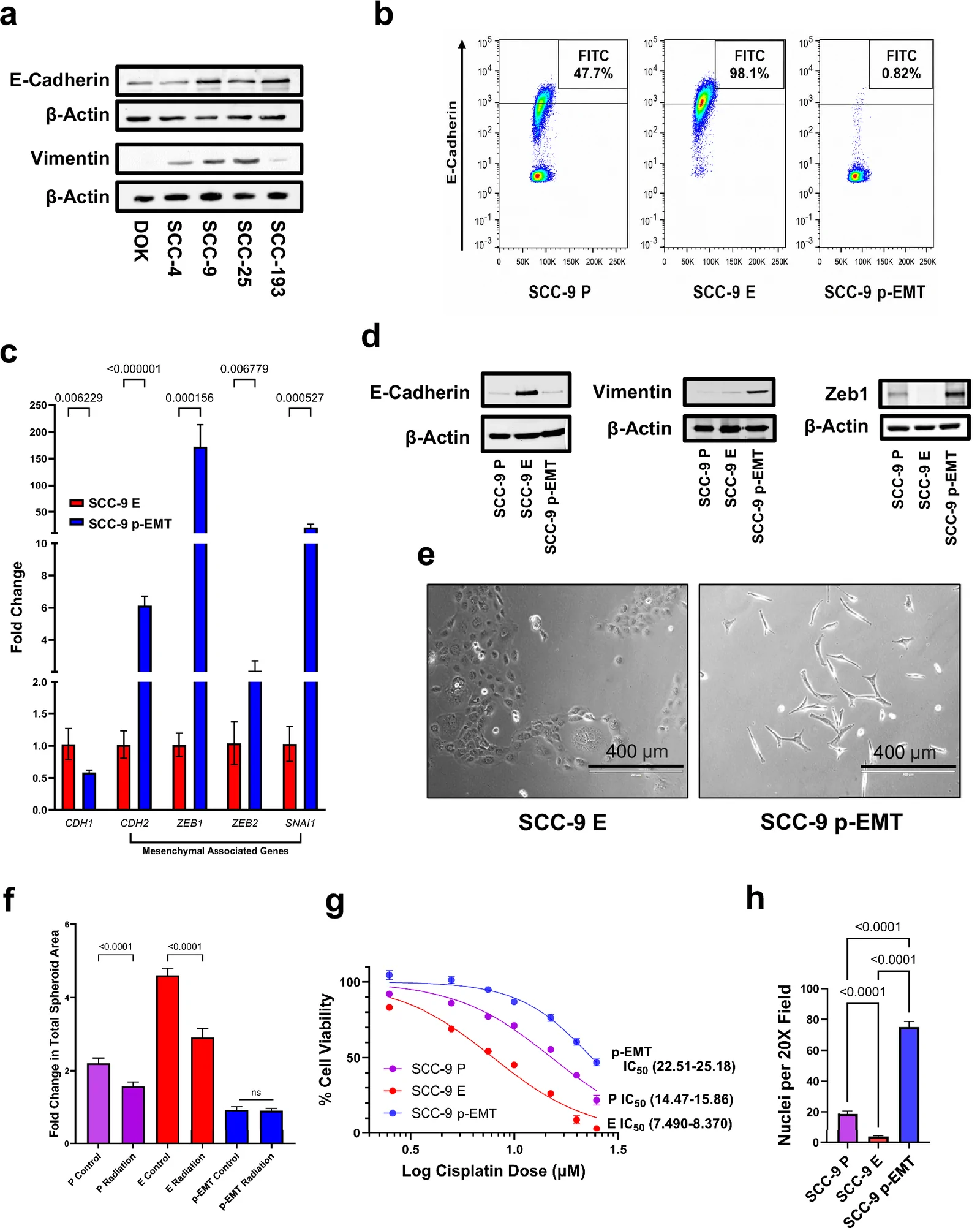
Establishing a model of OSCC EMT. **a** Immunoblots for E-cadherin and vimentin across dysplastic oral keratinocytes and four oral squamous cell carcinoma cell lines. **b** Flow cytometry diagrams of the parental SCC-9 line (SCC-9 P) and the derivative subpopulations, SCC-9 E (E-cadherin high, epithelial-like) and SCC-9 p-EMT (E-cadherin low, mesenchymal-like), after FACS. **c** Relative gene expression of five EMT related genes determined by RT-qPCR normalized to SCC-9 E baseline expression; *n* = 18, fold-change determined by 2^-ddCt^ method, error bars represent standard deviation, *p*-values above bars. **d** Immunoblots showing protein expression of E-cadherin, vimentin, and Zeb1 across SCC-9 P, E, and p-EMT cells. **e** Representative images of SCC-9 E and SCC-9 p-EMT subpopulations grown in culture. **f** Comparison of radiation resistance by measuring change in total spheroid area seeded with 10,000 cells one week after 4 Gy of ionizing radiation compared to control across SCC-9 P, E, and p-EMT cells; *n* = 16, error bars represent standard deviation, *p*-values between populations above bars. **g** Comparison of cisplatin IC_50_ curves across SCC-9 P, E, and p-EMT cells; *n* = 9, 95% CI in parentheses. **h** Results of 18-h Matrigel invasion assay comparing invasive potential between SCC-9 P, E, and p-EMT cells; *n* = 60, *p*-values between populations above bars.

To explore mechanisms of invasion in oral squamous cell carcinoma (OSCC), we first profiled canonical epithelial-to-mesenchymal transition (EMT) markers across dysplastic tongue-derived oral keratinocytes (DOKs) and multiple tongue-derived OSCC cell lines. Western blot revealed consistent E-cadherin expression in all lines, reflecting their epithelial origin, while vimentin levels were variably elevated in OSCC lines but absent in DOKs (Fig. 1a) (See Supplementary Information for Uncropped Immunoblots used in Figures). Notably, SCC-9 and SCC-25 lines displayed a high degree of EMT marker heterogeneity. This is in line with previous observations where SCC-25 cells were found to contain a subpopulation with high expression of stress response genes and SCC-9 cells a subpopulation with high expression of a partial-EMT (p-EMT) signature [9]. Prior efforts to isolate this p-EMT SCC-9 subpopulation confirmed its heightened invasive and resistant phenotype, but the cells rapidly reverted, reflecting the inherent metastability of p-EMT.

With several rounds of fluorescence-activated cell sorting on E-cadherin, we successfully isolated stable epithelial-like (SCC-9 E) and partial-EMT (SCC-9 p-EMT) subpopulations from the parental SCC-9 population (SCC-9 P) (Fig. 1b). These isogenic sublines retained their E and p-EMT identities over several weeks, serial passaging, and were confirmed to be genetically identical by short tandem repeat profiling (Supplementary Fig. 1). RT-qPCR confirmed a higher *CDH1* expression in SCC-9 E cells compared to SCC-9 p-EMT. SCC-9 p-EMT cells showed increased expression of *CDH2* and mesenchymal transcription factors *ZEB1*, *ZEB2*, and *SNAI1* (Fig. 1c), consistent with transcriptional EMT reprogramming. Gene changes were also reflected at the protein level (Fig. 1d). Morphologically, SCC-9 E cells exhibited a polygonal epithelial architecture, whereas SCC-9 p-EMT cells displayed a spindled, fibroblast-like phenotype (Fig. 1e).

Functionally, SCC-9 p-EMT cells demonstrated enhanced resistance to therapy and increased invasive capacity. Spheroid assays showed that SCC-9 E and P populations exhibited significant growth inhibition after exposure to ionizing radiation, while SCC-9 p-EMT cells were largely unaffected (Fig. 1f and Supplementary Fig. 2). Similarly, cisplatin dose-response curves revealed a higher IC₅₀ in SCC-9 p-EMT cells compared to SCC-9 E and P subpopulations (Fig. 1g). In Matrigel invasion assays, SCC-9 p-EMT cells were significantly more invasive than either SCC-9 E or P (Fig. 1h), and wound healing assays confirmed enhanced migratory behavior (Supplementary Fig. 3). Together, these data establish SCC-9 E and p-EMT as stable, transcriptionally and phenotypically distinct subpopulations that define an OSCC p-EMT spectrum. This model provides a robust in vitro platform to dissect metastable molecular regulators associated with aggressive clinical behaviors such as increased invasion and therapeutic resistance.

### OSCC p-EMT correlates with increased expression of an invasion-permissive ECM

**Fig. 2:**
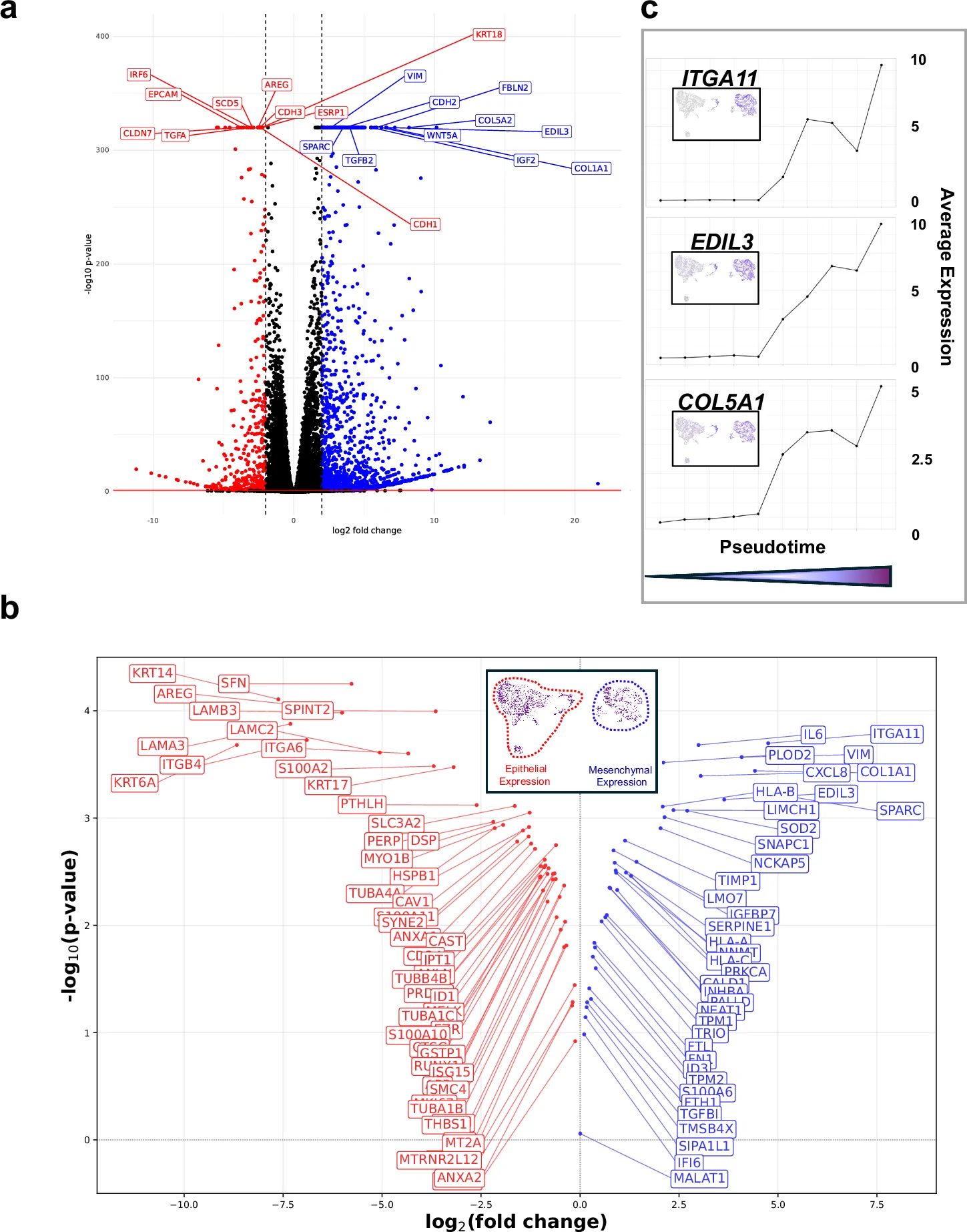
Transcriptional differences between SCC-9 E and SCC-9 p-EMT. **a** Volcano plot of the most statistically significant differentially expressed genes (DEGs) between SCC-9 E and SCC-9 p-EMT cells as determined by bulk RNA sequencing. All DEGs have at least a twofold change in expression between cell subpopulations. **b** Differential gene expression determined by single-cell RNA sequencing. The inset shows the SCC-9 E cluster (epithelial expression) and SCC-9 p-EMT cluster (mesenchymal expression) derived from these data. **c** Pseudotime projections of the top three genes most associated with the mesenchymal expression cluster determined by Moran’s I statistic of single-cell RNA sequencing data. Left-to-right corresponds to epithelial-to-mesenchymal gene expression changes along pseudotime.

Consistent with EMT-associated reprogramming, we observed broad gene expression differences, particularly among components of the extracellular matrix (ECM) between SCC-9 E and SCC-9 p-EMT subpopulations. Compared to SCC-9 p-EMT cells, SCC-9 E cells significantly upregulated genes involved in maintaining epithelial identity, cell–cell adhesion, and tight junction integrity, such as *CDH1*, *ESRP1*, *AREG*, *ITGA6*, and *CLDN7* [13–16] (Fig. 2a). In contrast, SCC-9 p-EMT cells strongly upregulated genes associated with a mesenchymal-like ECM composition, including *CDH2*, *WNT5A*, *COL1A1*, *COL5A2*, and *EDIL3* [17–19]. SCC-9 p-EMT cells also upregulated several genes previously used to define a p-EMT state in OSCC, including *VIM*, *MMP1/2/3*, and *SERPINE1* [9]. The switch from high expression of *CDH1* to *CDH2*, elevated *VIM*, and increased fibrillar collagen transcripts reflected a transition to a more invasive phenotype. *EDIL3*, which codes for a secreted glycoprotein that binds integrin α_5_/α_v_ to activate FAK signaling, was also sharply upregulated in p-EMT cells, suggesting a potential role in OSCC invasion.

To further resolve EMT dynamics, we performed single-cell RNA sequencing on SCC-9 P, E, and p-EMT cells. Unsupervised clustering revealed transcriptionally distinct subpopulations (inset of Fig. 2b): E cells clustered tightly with epithelial markers, p-EMT cells formed distinct mesenchymal-like clusters, and P cells contained populations that fell into either of the two states. Differential expression analysis comparing E and p-EMT clusters highlighted upregulation of *KRT6A*, *KRT14*, *AREG*, and *ITGB4* in E cells, and *SPARC*, *ITGA11*, *COL1A1*, *VIM*, and *EDIL3* in p-EMT cells, further reinforcing their divergent identities [20–22] (Fig. 2b).

Pseudotime analysis ordered cells along a clear epithelial-to-mesenchymal trajectory, with epithelial markers enriched early and mesenchymal markers including *ITGA11* (an integrin associated with increased invasion that binds fibrillar collagens), *EDIL3*, and the fibrillar collagen gene *COL5A1* rising later in the transition (Fig. 2c). This trajectory captures the emergence of the p-EMT state, where partial activation of mesenchymal programs coincides with increased invasion-associated signaling. The sharp increase in *EDIL3* along this trajectory, coupled with its low baseline expression in normal oral mucosa (Supplementary Fig. 4), strongly supports its association with p-EMT and its potential as a biomarker of OSCC invasion. While EDIL3 has been implicated in driving EMT in other cancers, its functional role in OSCC remains largely unexplored.

### Differential chromatin accessibility highlights p-EMT-associated regulatory programs

**Fig. 3:**
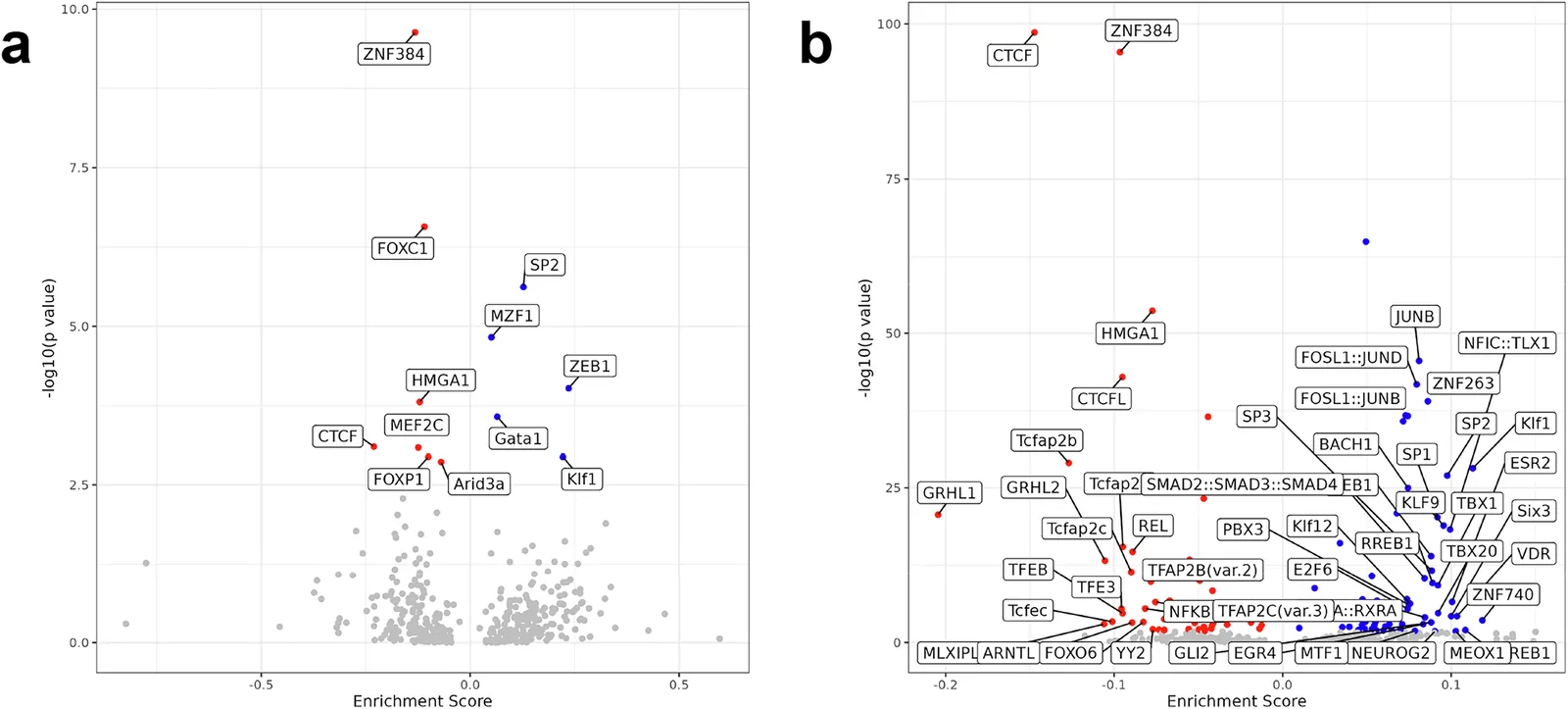
Differences between SCC-9 E and p-EMT cell chromatin accessibility. **a** Transcription factor motif enrichment at promoter ATAC-sequencing peaks for SCC-9 E (red) and SCC-9 p-EMT (blue) cells. **b** Transcription factor motif enrichment at distal intergenic ATAC-sequencing peaks for SCC-9 E (red) and SCC-9 p-EMT (blue) cells.

The striking phenotypic and gene expression differences between SCC-9 E and SCC-9 p-EMT cells despite their genetic equivalence suggest substantial epigenetic modulation. To investigate this, we performed ATAC-seq to compare chromatin accessibility between epithelial and p-EMT states. Differential accessibility analysis revealed widespread divergence in accessible chromatin. KEGG pathway analysis of differentially accessible promoter regions showed enrichment in cancer-associated signaling pathways. SCC-9 p-EMT cells exhibited greater promoter accessibility at genes involved in MAPK signaling, TGF-β signaling, WNT signaling, ECM–receptor interaction, and actin cytoskeleton regulation, including pro-migratory and p-EMT-associated genes such as *MAPK3*, *PAK1*, *TGFBR2*, *FGFR1*, *PDGFRA*/*B*, *ITGA5*, *ITGAV*, and *ITGB3*, as well as cytoskeletal regulators like *ACTN1*, *ACTN4*, and *TIAM1* [23–26] (Fig. 3a). In contrast, SCC-9 E cells showed greater accessibility at promoters of epithelial adhesion and polarity genes, including *CDH1*, *CTNNA1*, *CLDN1*, *EPCAM*, and *DSG3* [14, 27].

Enhancer-level analysis revealed even broader regulatory differences. SCC-9 p-EMT cells showed increased accessibility at enhancers linked to focal adhesion, cytokine and growth factor signaling, and transcriptional elongation, including regulators of the SWI/SNF complex (*SMARCC2*, *SMARCD3*) [28] (Fig. 3b). Conversely, SCC-9 E-specific enhancers remained accessible at canonical transcriptional repressors and chromatin insulators such as *EZH2*, *YY1*, *RBBP7*, *HDAC1*, and *CTCF*, which may help reinforce epithelial identity [29].

Transcription factor (TF) motif enrichment further highlighted a divergence in regulatory programs. SCC-9 p-EMT promoter peaks were enriched for motifs associated with EMT and lineage plasticity, like *ZEB1*, *KLF1*, *SP2*, *GATA1*, and *MZF1* [30]. In contrast, SCC-9 E promoter peaks favored motifs for *HMGA1*, *CTCF*, *MEF2C*, and *FOXP1*, factors associated with chromatin structure and transcriptional insulation [29, 31].

At enhancers, SCC-9 p-EMT cells were enriched for motifs of EMT-associated regulators such as *ZEB1*, *SMAD2*/*3*/*4*, *FOS::JUN*, *GLI2*, *KLF13*, and *EPAS1* [32], whereas SCC-9 E-specific enhancers retained accessibility at motifs for *GRHL1/2*, the *TFAP2* family, *FOXP1*, and *CTCF*, all of which promote epithelial polarity and adhesion [33].

Together, these data reveal a clear epigenetic divide between epithelial and p-EMT OSCC states. p-EMT cells maintain accessibility at regulatory elements associated with motility and invasion while epithelial cells preserve accessibility at loci reinforcing structural identity. This chromatin landscape suggests that both states exist in epigenetically dynamic configurations poised to support epithelial or mesenchymal transcriptional programs, providing an epigenetic basis for phenotypic plasticity in OSCC.

### Critical drivers of p-EMT-driven invasion center around cytoskeletal rearrangement and cell adhesion

**Fig. 4:**
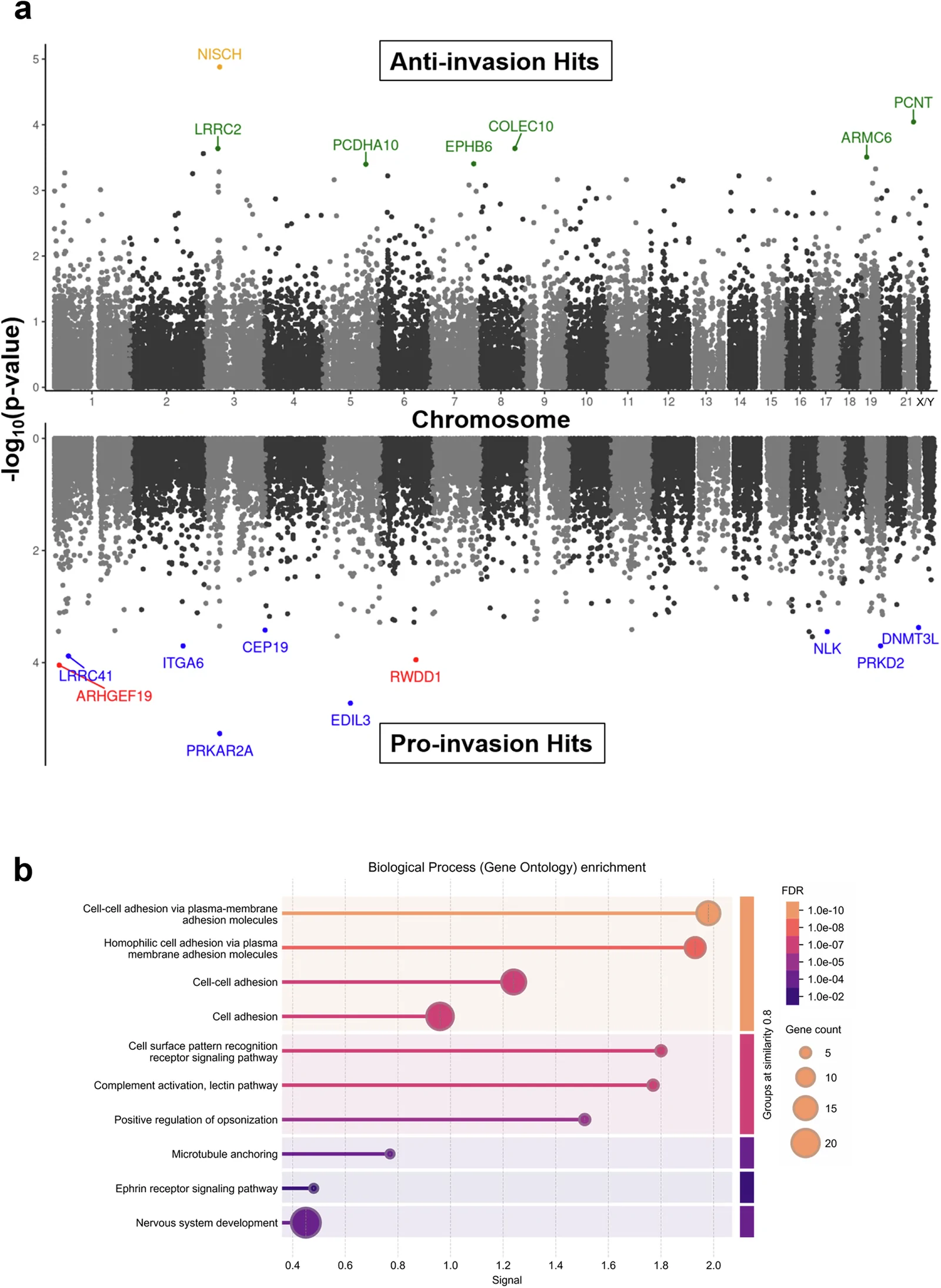
Identification of causal drivers of OSCC invasion using genome-wide CRSIPR screening. **a** 17 pro- and anti-invasion modulators were identified by comparing sgRNA enrichment between invaders and non-invaders (blue and green) and between descenders and non-invaders (red and yellow). **b** Gene Ontology Analysis of Biological Processes on all 17 identified invasion modulators.

The data suggest that SCC-9 E and p-EMT cells represent both ends of an OSCC p-EMT spectrum. However, the gene expression changes identified so far remain correlative. To pinpoint causal drivers of OSCC invasion, we performed a novel genome-wide CRISPR knockout screen leveraging the power of the SCC-9 p-EMT phenotype to interrogate both pro- and anti-invasion regulators. Briefly, constitutive-Cas9-expressing SCC-9 p-EMT cells were transduced with a genome-scale lentiviral sgRNA library and subjected to invasion assays on basement membrane extract. Following invasion, non-invading cells in the upper chamber were isolated and separated from cells that invaded into the lower chamber. DNA was collected from each group, and sgRNA abundance was assessed by deep sequencing (Fig. 4a).

Seventeen statistically significant invasion-modulating genes were identified (Fig. 4b). The ten pro-invasion hits were: *ARHGEF19*, *RWDD1*, *PRKAR2A*, *EDIL3*, *LRRC41*, *ITGA6*, *PRKD2*, *NLK*, *CEP19*, and *DNMT3L*. Seven anti-invasion genes were also identified: *NISCH*, *PCNT*, *LRRC2*, *COLEC10*, *ARMC6*, *EPHB6*, and *PCDHA10* (Fig. 4c). Importantly, this functional screen uniquely enabled identification of genes required for the transient cellular state of active invasion, regulators that are unlikely to emerge from genomic analyses alone because they are not recurrently mutated but instead dynamically deployed.

Network analysis using STRING revealed two major clusters (Supplementary Fig. 5). A cytoskeletal reorganization group of genes implicated in microtubule organization, centrosome localization, RhoA signaling, and actin remodeling (e.g., *ARHGEF19*, *PRKAR2A*, *PRKD2*, and *CEP19*) [34–39]. Additionally, a cell adhesion group featured genes related to integrin signaling and cell junctional stability, including *EDIL3*, *ITGA6*, *COLEC10*, *EPHB6*, and *NISCH* [15, 40–44]. The identification of *DNMT3L*, a DNA methylation modulator and regulator of DNMT3A/B, as a pro-invasion hit further reinforces the need for epigenetic remodeling to maintain the invasive, p-EMT state in SCC-9 cells [45–47]. Comparison of sgRNA abundance in the baseline-transfected library versus the SCC-9 p-EMT cells used in the screen revealed a strong EZH2 and DNMT3 signature required for maintenance of the p-EMT state, corroborating our ATAC-seq findings and further implicating DNMT3L as a driver of OSCC p-EMT and invasion (Supplementary Fig. 6).

Interestingly, *ITGA6*, which is more highly expressed in the SCC-9 E subpopulation (Fig. 2a), appears to be required for invasion. This supports a model in which this ECM receptor aids in binding to the basement membrane not to maintain epithelial barrier integrity but rather to facilitate invasion, an established context-dependent function. Importantly, this screen provides the first direct evidence that *EDIL3* is not merely strongly correlated with p-EMT and OSCC invasion but acts as a causal driver of the phenotype and is actively required for invasion. Furthermore, the identification of *NISCH*, a known inhibitor of EDIL3-mediated FAK signaling, as an anti-invasion hit further highlights a regulatory axis in which EDIL3 signaling is a necessary but modifiable driver of OSCC invasion [40, 48]. Together, these data show that OSCC invasion requires coordinated cytoskeletal, adhesion, and epigenetic regulators. Our stabilized p-EMT model, combined with an unbiased screen, uncovered modulators normally appreciable only during the dynamic, metastable process of invasion.

### EDIL3 drives p-EMT, invasion, and therapeutic resistance

**Fig. 5:**
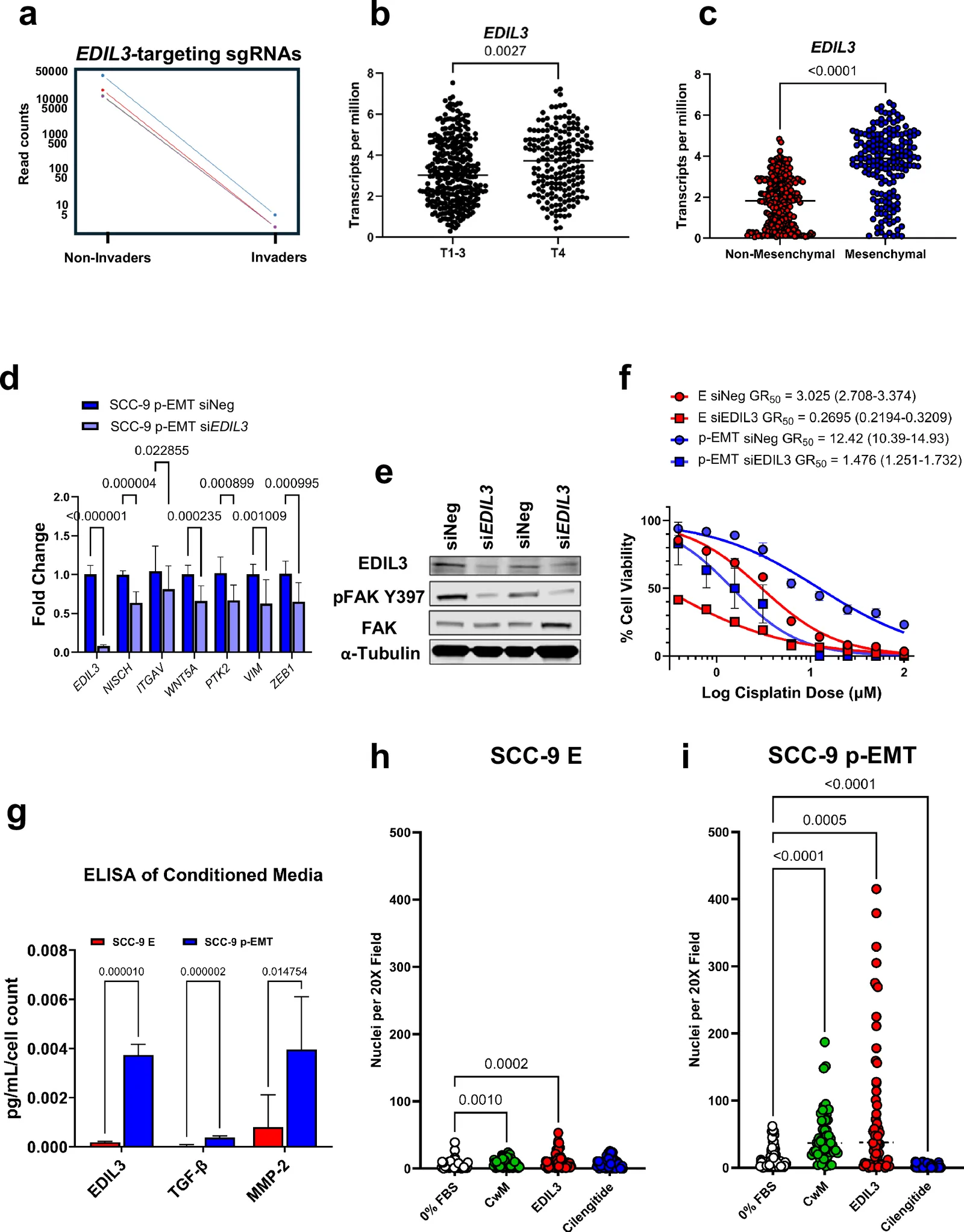
EDIL3 drives OSCC EMT, cisplatin resistance, and invasion. **a** Enrichment of unique, *EDIL3*-targeting sgRNAs in the knockout CRISPR screen described in Fig. 4. All four were much more highly enriched in non-invaders compared to invaders, identifying *EDIL3* as a major causal driver of OSCC invasion. **b** *EDIL3* mRNA expression of pT1-T3 tumors from TCGA-HNSCC dataset compared to pT4 tumors. **c** Comparison of *EDIL3* mRNA expression between mesenchymal and non-mesenchymal subtypes of tumors within the TCGA-HNSCC dataset. **d** Gene expression changes of EMT-related genes in SCC-9 p-EMT cells after 48 h of *EDIL3* knockdown using siRNA. The numbers above bars represent *p*-values, *n* = 18. **e** Immunoblot of two biological replicates of SCC-9 p-EMT cells after 72 h of *EDIL3* knockdown with siRNA. Loss of EDIL3 expression corresponds to a loss of FAK activation. **f** Cisplatin GR_50_ curves for SCC-9 E and SCC-9 p-EMT cells after 48 h *EDIL3* siRNA knockdown. **g** Protein concentration for EDIL3, TGF-β, and MMP-2 in media conditioned for 24 h as measured by ELISA. EDIL3 was above the maximum detection limit in SCC-9 p-EMT conditioned media, therefore, this maximum is what is represented graphically; *n* = 6, error bars represent standard deviation, *p*-values above bars. **h** SCC-9 E cell invasion through 10% Matrigel at 18 h. Data represent five nuclei counts per 20× powered field per Transwell insert. Numbers above brackets correspond to *p*-values between comparisons. **i** Same experiment and data representation as in (**h**) but for SCC-9 p-EMT cells.

*EDIL3* was consistently identified as a top p-EMT gene across bulk and single-cell RNA sequencing, pseudotime analysis, and emerged as a major pro-invasion driver in our CRISPR screen, where its inhibitor *NISCH* was also identified as an anti-invasion gene (Figs. 2–4 and 5a).

Though we argue that EDIL3 plays a vital but transient role in OSCC invasion, we reasoned that larger, more invasive, and more mesenchymal tumors may show increased total *EDIL3* expression due to a larger proportion of cells actively expressing the gene. To test this, we analyzed patient tumor data from the TCGA-HNSC cohort. As predicted, *EDIL3* mRNA levels were significantly elevated in head and neck squamous cell carcinomas that invade into adjacent structures (pT4) compared to tumors restricted to their tissue of origin (pT1–T3) (Fig. 5b), reinforcing the association of *EDIL3* with a more aggressive phenotype. Likewise, *EDIL3* expression was markedly higher in mesenchymal tumors relative to classical, basal, or atypical gene expression subtypes (Fig. 5c).

To validate the causal contribution of *EDIL3* on OSCC EMT, we silenced its expression using siRNA in SCC-9 E and p-EMT cells. In SCC-9 p-EMT cells, *EDIL3* knockdown significantly reduced expression of several mesenchymal-associated genes, including *VIM*, *ZEB1*, *WNT5A*, *ITGAV*, and *PTK2* (FAK), while E cells were less affected (Fig. 5d, and Supplementary Fig. 7). These changes support a model in which *EDIL3* promotes and sustains the p-EMT state in OSCC. This is reflected at the protein level as well where *EDIL3* knockdown leads to a loss of FAK activation (Fig. 5e). We next evaluated whether EDIL3 modulates therapy response as it has been shown to promote paclitaxel resistance in breast cancer [48]. siRNA-mediated *EDIL3* knockdown sensitized both SCC-9 E and p-EMT cells to cisplatin, reducing the GR_50_ by approximately 10-fold in both E and p-EMT cells (Fig. 5f). These findings suggest that *EDIL3* also contributes to chemoresistance across OSCC EMT states.

As EDIL3 is a secreted protein [49], we used ELISA to measure levels in E and p-EMT conditioned media. EDIL3 levels also positively correlated with expression of mesenchymal proteins across multiple OSCC cell lines (Supplementary Fig. 7). We next showed that p-EMT-conditioned media rich in EDIL3 (Fig. 5g) increased invasion of both SCC-9 E and SCC-9 p-EMT cell states (Fig. 5h, i). Since p-EMT-conditioned media is also rich in other EMT factors like TGF-β and MMP2, we showed that recombinant EDIL3 on its own enhanced invasion of both subpopulations. Inversely, blocking integrin α_v_ with cilengitide [50] abrogated invasion, confirming EDIL3’s dependence on this pathway [48].

### p-EMT invasion signature defines the invasive front of OSCC

**Fig. 6:**
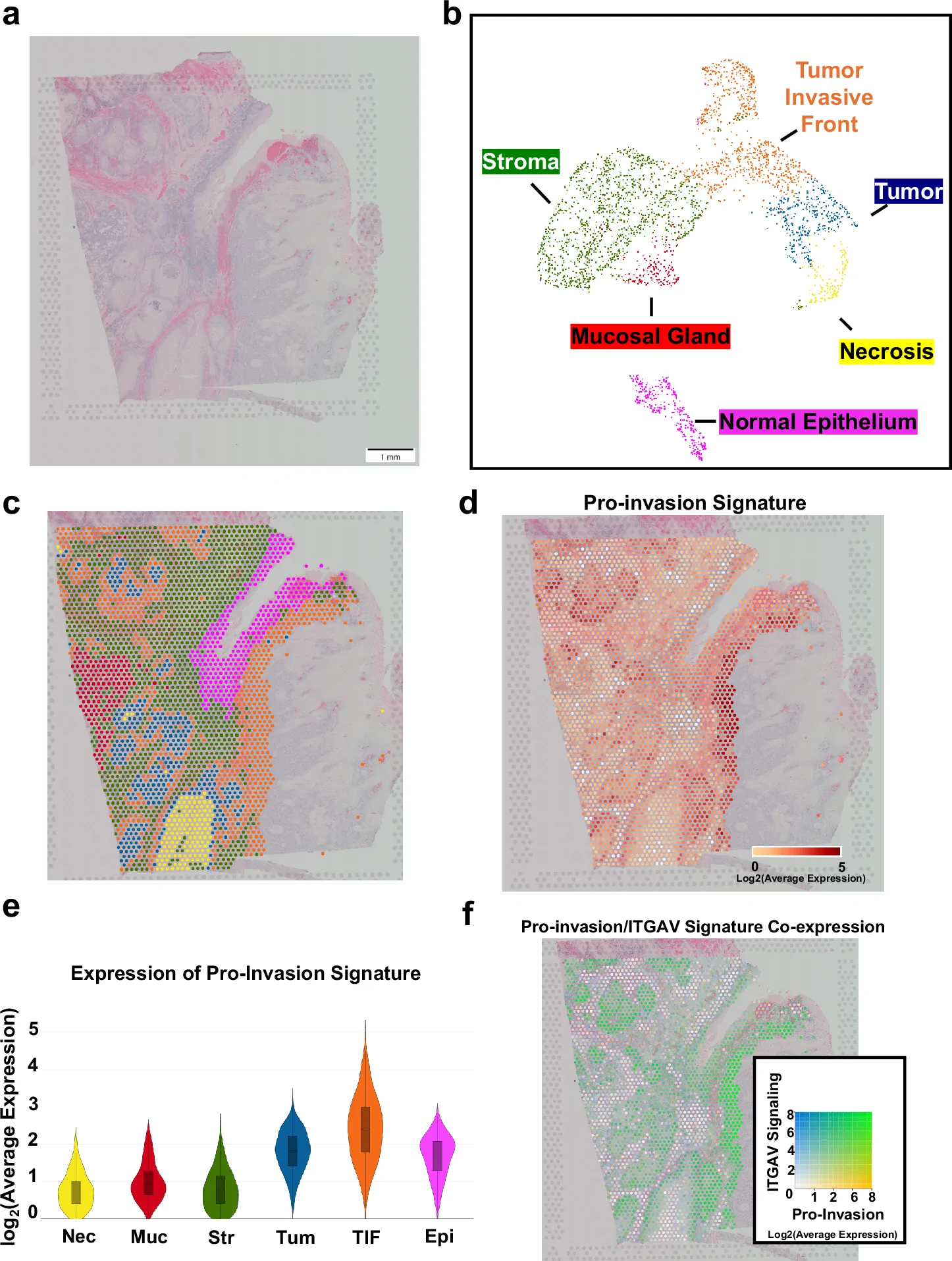
OSCC pro-invasion gene signature identifies invasive tumor front. **a** H&E staining of OSCC tissue sample used for spatial transcriptomic analysis. **b** Unsupervised clustering of gene expression signatures. **c** Cluster colors overlayed on tissue sample. **d** Average expression of pro-invasion genes identified in CRISPR screen is highest at tissue regions that correspond to the tumor invasive front. **e** Graphical representation of data from (**d**). Nec Necrosis, Muc Mucosal Gland, Str Stroma, Tum Tumor, TIF Tumor Invasive Front, and Epi Normal Epithelium. **f** Co-expression of pro-invasion signature and integrin *α*_v_ signaling genes (*ITGAV, ITGB1, ITGB3, PTK2, FN1, SPP1*, and *EDIL3*). Blue regions indicate high integrin α_v_ signature, yellow regions indicate high pro-invasion signature, green regions indicate high co-expression.

To assess clinical relevance, we performed spatial transcriptomic profiling on a recurrent OSCC specimen (Fig. 6a), annotated into tumor, stroma, and epithelium for spatial mapping (Supplementary Fig. 8). Canonical EMT genes (*VIM*, *FN1*, *TWIST2*, *ZEB2*) localized near the tumor–stroma interface, consistent with a transcriptionally definable invasive front (Supplementary Fig. 9). To explore this directly, we performed unsupervised clustering and identified transcriptionally distinct regions aligning with histological compartments and the tumor invasive front (Fig. 6b, c).

Tumor regions expressed epithelial transcripts (*MUC1*, *KRT16*, *LY6D*, *LCN2*, *SAA1*, kallikreins) [51–56] and immune-modulatory genes (*CXCL8*, *S100A8*/*A9*) [57, 58]. This transcriptional profile is consistent with either a classical or basal molecular subtype, marked by squamous differentiation, epithelial lineage commitment, and immune infiltration. The invasive front showed modest upregulation of wound-healing genes (e.g., *FBN2*) [59] and broad downregulation of epithelial differentiation (*MUC1*, *ELF3*, *SCNN1A*) [60, 61] and innate immune genes (*S100A7*, *DEFB4A*, *CXCL17*) [62–64], suggesting partial epithelial loss.

Strikingly, applying our unbiasedly derived p-EMT pro-invasion gene signature to the spatial transcriptomic data robustly delineated the tumor invasive front (Fig. 6d, e). Key contributors included *ITGA6*, *RWDD1*, and *NLK*, reflecting the adhesion and cytoskeletal pathways identified in our functional network analysis. Although *EDIL3* itself was expressed at low levels, consistent with its minimal expression in normal oral mucosa (Supplementary Fig. 4). Downstream partners (*ITGAV*, *ITGB1/3*, *PTK2*) and their alternate substrates (*FN1*, *SPP1*) [65] were enriched at the invasive front and correlated with the pro-invasion signature (Fig. 6f). In contrast, *WNT5A*, a non-canonical Wnt ligand, upstream activator of RhoA signaling, and EMT factor [19] also differentially upregulated in SCC-9 p-EMT cells (Fig. 2a), exhibited high expression in both tumor and normal oral epithelium, limiting its tumor specificity (Supplementary Fig. 10). This suggests that integrin α_v_ signaling may be a critical nexus in driving OSCC EMT and invasion. Given the association of EDIL3 with integrin α_v_ signaling, it may drive the p-EMT cell state and associated invasion despite modest expression, a key insight only made possible through our approach of combining functional screening and genomics.

In summary, the p-EMT pro-invasion network is recapitulated in spatially resolved OSCC tissue, underscoring the therapeutic relevance of integrin α_v_ signaling as both a biomarker and target, exploitable by cilengitide or FAK inhibitors.

## Discussion

Oral squamous cell carcinoma (OSCC) is an aggressive malignancy with high recurrence, therapeutic resistance, and few targetable mutations. Progression often occurs against a similar genetic background, complicating efforts to pinpoint drivers of invasion. To address this, we developed an isogenic p-EMT OSCC model and conducted a genome-wide CRISPR invasion screen, integrating transcriptomic, epigenomic, and spatial profiling to uncover a targetable invasion network. These genes converge on two main interconnected processes: cytoskeletal reorganization and cell adhesion, with epigenetic remodeling underlying both. Unlike expression-based analyses, our functional screen directly identified the requirement of specific genes for invasion, effectively distinguishing correlative markers from true effectors.

Among the 17 high-confidence hits, pro-invasion genes including *EDIL3*, *ITGA6*, and *ARHGEF19* aligned with known drivers of cytoskeletal and adhesion-based invasion, while anti-invasion genes such as *NISCH* and *EPHB6* support barrier stabilization and suppress motility [34, 42–44, 48]. The identification of *DNMT3L* and epigenetic regulatory signatures highlights the role of chromatin remodeling in enabling the mesenchymal-like transcriptional state [45–47]. The localization of the screen-derived p-EMT pro-invasion signature to the tumor invasive front of OSCC and its significant co-expression with genes involved in integrin α_v_ signaling suggests a common signaling axis utilized by OSCC cells during invasion.

Several pro-invasion hits identified in our screen, including *ITGA6* and *EDIL3*, converge on integrin–FAK signaling, an axis repeatedly implicated in tumor cell invasion. Integrin α_6_, a core hemidesmosome component, has been associated with poor prognosis in multiple squamous carcinomas and can promote invasion via FAK activation [15]. Anti-invasion hits, including EPHB6 and PCDHA10, emphasize the dual potential for modulation within adhesion machinery [66]. Additional hits, such as *ARHGEF19* and *NLK* involved in RhoA signaling [34, 67], and vesicle trafficking regulators *PRKAR2A*, *RWDD1*, and *PRKD2*, highlight roles for actin remodeling and cargo delivery in OSCC invasion [35–37, 68]. Together, these pathways support a model in which adhesion, cytoskeletal dynamics, and vesicle trafficking collectively drive invasion.

*EDIL3* emerged as a major OSCC invasion driver. Not only was EDIL3 significantly upregulated in the SCC-9 p-EMT cells compared to the epithelial SCC-9 E cells based on bulk RNA sequencing, but its expression also increased sharply along the EMT spectrum. *EDIL3* was independently identified as a pro-invasion driver in our genome-wide screen, while its inhibitor *NISCH* was identified as an anti-invasion driver within the same screen. Follow-up functional assays showed that *EDIL3* knockdown impaired chemoresistance and the expression of mesenchymal genes. Additionally, we showed that EDIL3 secretion was highly SCC-9 p-EMT specific, enhances invasion, and that inhibition of its receptor integrin α_v_ with cilengitide abrogates this effect. Furthermore, spatial transcriptomics localized the p-EMT pro-invasion signature and integrin α_v_ signaling to the invasive front of aggressive OSCC. As EDIL3 is an actively secreted protein, it is likely that it is acting as an autocrine and/or paracrine factor to maintain the pro-invasive state and may explain why we were able to isolate a stable SCC-9 subpopulation that shares many characteristics with metastable p-EMT OSCC cells.

In conclusion, our findings support a model in which a complex, dynamic, and often transient metastable state of mesenchymal-like cells drives aggressive characteristics such as invasion and therapeutic resistance in OSCC. This mesenchymal-like state, exemplified by SCC-9 p-EMT cells, requires coordinated cytoskeletal, adhesion, and epigenetic programs to drive invasion. The functional convergence of these pathways on the invasive front of human tumors supports their relevance and nominates EDIL3–integrin α_v_ signaling as a promising novel therapeutic vulnerability. As further uses for cilengitide are explored and FAK-targeting agents advance clinically, our work provides a mechanistic rationale for testing these approaches in clinical trials for patients with OSCC.

## Methods

### Cell lines and cell culture

Human OSCC cell lines SCC-4 (RRID:CVCL_1684), SCC-9 (RRID:CVCL_1685), and SCC-25 (RRID:CVCL_1682) were obtained from the American Type Culture Collection (ATCC, Manassas, VA). The OSCC cell line SCC-193 (RRID:CVCL_1288) and the human dysplastic oral keratinocyte line, DOK (RRID:CVCL_1180), were obtained from a colleague (Dr. Liu Hong, University of Iowa). Cell lines were cultured using DMEM-F12 media (Gibco; Thermo Fisher Scientific, Inc., Waltham, MA) supplemented with 10% fetal bovine serum (FBS; Biowest, Bradenton, FL) and 1X GlutaMAX Supplement (Gibco; Thermo Fisher Scientific, Inc., Waltham, MA.). Media also contained 1X penicillin/streptomycin unless used for transfection. All cell lines were cultured in a humidified incubator at 37 °C with 5% CO2. The cell lines were validated with short tandem repeat profiling and tested for mycoplasma contamination (IDEXX Laboratories, Inc., Westbrook, ME). Cell images were taken on an EVOS fl fluorescent microscope (Thermo Fisher Scientific, Inc., Waltham, MA).

### Immunoblotting

OSCC cells were plated at 250,000 cells/well in 6-well plates and incubated for 48 h. Cells were harvested by cell scraper (or 0.25% trypsin/Versene solution for Fig. 1D) and whole cell lysates made with 200 mL lysis buffer (4× Laemmli Sample Buffer, Biorad, Hercules, CA). Between 20–40 mg of protein was separated on SDS-polyacrylamide gradient gels (NuPAGETM, Thermo Fisher Scientific, Waltham, MA) and transferred to a PVDF membrane (Immobilon-FL, EMD Millipore, Burlington, MA) overnight at 4 °C. Membranes were blocked for 1 h at room temperature (Intercept Blocking Buffer, LI-COR, Lincoln, NE), washed with 1X phosphate-buffered saline, and incubated in primary antibodies for 1 h at room temperature. Primary antibodies utilized include: Vimentin (1:1000, Developmental Studies Hybridoma Bank, Iowa City, IA), Pan Cytokeratin (MA5-13203, 1:1000, Invitrogen, Waltham, MA), E-Cadherin (AF748, 1:1000, R&D Systems, Minneapolis, MN), ZEB1 (NBP1-05987, 1:1000, NOVUS Bio, Centennial CO), b-Actin (BA3R, 1:2000, Invitrogen, Waltham, MA), FAK (3285, 1:1000, Cell Signaling Technology, Boston, MA), Phospho-FAK (Tyr397) (D20B1) (8556, 1:1000, Cell Signaling Technology, Boston, MA), and EDIL3 (1:1000, Proteintech, Rosemont, IL). Membranes were washed in 1× PBS and then incubated with appropriate fluorescent secondary antibodies (1:10 000, LI-COR, Lincoln, NE) and scanned using an Odyssey Imaging System (LI-COR, Lincoln, NE).

### Transwell invasion assays

12-well plates with Transwell membrane inserts containing 8.0 mm membrane pores (Corning, Glendale, AZ) were coated with 40 mL 10% Matrigel Matrix (Corning, Glendale, AZ) diluted in serum-free media and incubated for 30 min at 37 °C. Cells were plated at a density of 100,000 cells/well in 100 mL serum-free media in the top chamber, and 20% FBS-containing media was added to the bottom chamber. After 18 h, cells were gently removed from the upper chamber membrane using a cotton tip applicator x3, and invaded cells were fixed and stained using 4,6-diamidino-2-phenylindole (DAPI) fluorescent nuclear stain (ThermoFisher Scientific). Invaded cells were quantified by counting DAPI-positive cells per 20X field, 5 fields per well. Conditioned media was collected from T175 flasks containing SCC-9 p-EMT cells at 80% confluence after 24 h of incubation in fresh media. For EDIL3-containing media, Recombinant Human EDIL3 Protein, CF (Bio-Techne, Minneapolis, MN) was added at a concentration of 5 μg/mL. For cilengitide-containing media, Cilengitide (Bio-Techne, Minneapolis, MN) was added for a final concentration of 10 μM.

### Flow cytometry and sorting

Fluorescence-activated cell sorting (FACS) analysis was performed using fluorescein (FITC) dye (1:100, Millipore, Burlington, MA), E-cadherin antibody (1:33, R&D Systems, Minneapolis, MN), and Integrin beta 4 antibody (1:20, Invitrogen, Waltham, MA). Matched isotype was used as a negative control (IgG1 from murine myeloma, 1:100, Millipore, St. Louis, MO). Versene solution (Thermo Fisher Scientific, Inc., Waltham, MA) was used to collect 300,000 cells per sample. Cells were rinsed and then resuspended in 100 μL FACS buffer. Primary antibody or isotype alone was then added to the respective samples and incubated for 45 min at 37 °C. Cells were rinsed and incubated with goat anti-mouse secondary antibody (Gt x Ms IgG FLUOR FITC, 1:1000, Millipore, St. Louis, MO) in FACS buffer (1 L DPBS, 10 g BSA, 1 g sodium azide) for 45 min at 37 °C, followed by a final FITC incubation for 45 min at 37 °C. Cells were then washed, resuspended in FACS buffer, and 5 μg/mL Hoechst was added to determine cell viability (Millipore Sigma, Burlington, MA). FACS analysis was performed on a Becton Dickinson LSR II Flow Cytometer with violet laser. FlowJo software was utilized to analyze data.

For sorting and subculturing of the SCC-9 parental line, 2 × 10^6^ cells were used per sample. After incubation with appropriate primary and secondary antibodies, cells were passed through a 70 μm mesh filter and collected in 700 μL FACS buffer. E-Cadherin positive and E-Cadherin negative cells were sorted with assistance of the University of Iowa Flow Cytometry Core, collected into tissue culture media, and plated into 12-well plates for incubation and further passaging.

### Radiation sensitivity

Cultrex UltiMatrix Reduced Growth Factor Basement Membrane Extract (Bio-Techne, Minneapolis, MN) was thawed overnight at 4 °C. 96-well, black-bordered cell culture plates were brought to 37 °C overnight. Cells were collected, washed with DPBS, and resuspended in ice-cold Basement Membrane Extract at either 5000 or 10,000 cells per 5 µL. We then pipetted 5 µL of the suspension into each of the inner-most 60 wells. Plates were carefully inverted and placed at 37 °C for 20 min to allow for dome formation and polymerization. The plates were placed right side up for another 20 min at 37 °C then covered with 200 µL of 10% FBS containing F12/DMEM media with penicillin/streptomycin. Positive controls included staurosporine (*n* = 2) at a concentration of 500 nM and experimental groups (*n* = 8) contained a matching volume of vehicle (DMSO). Radiation-treated cells received 4 Gray on Day 0 using cesium-137-generated gamma rays using the Radiation and Free Radical Research Core of the University of Iowa. Spheroid formation was monitored using a Cytation 5 (Agilent/Biotek) with a 4X objective and bright field and fluorescent (TRITC) channels, with 15 images acquired for each well along the Z-plane at ~100 µm intervals [69]. Gen5 software (Agilent, Santa Clara, CA) was used to transform Z-plane images to obtain Z-projections for each well that captured each dome’s entirety. The masking threshold was set between 20 and 1000 µm to quantify spheroids. If necessary, the upper limit was adjusted to capture larger spheroid structures.

### Cisplatin sensitivity assays

For baseline calculations, cells were plated at 10,000 cells/well in 12-well plates. After 24 h cells were treated with 0–25 μM cisplatin (TargetMol, Wellesley Hills, MA) diluted in 1× PBS. Cells were incubated in PBS vehicle only or cisplatin for 72 h after which viability was assessed using a Resazurin reduction assay (Sigma-Aldrich, St. Louis, MO). 1X Resazurin was added to cells at a concentration of 0.025 mg/mL and incubated for 2 h at 37 °C. A Synergy HT plate reader (Biotek, Winooski, VT) was used to read fluorescence with excitation at 560 nm and emission at 590 nm. Experimental values were normalized to the PBS vehicle control for each cell line. The absolute IC_50_ was calculated using GraphPad Prism 9.0 software (GraphPad Software, Boston, MA).

For siRNA calculations, cells were plated at 2500 cells/well in 96-well plates containing preformed siRNA (5 pmol) and Lipofectamine 2000 (Invitrogen, Waltham, MA) complexes. At 48 h, old media was replaced with 100 μL of media-diluted cisplatin at concentrations between 0 and 120 μM. At 96 h, 20 μL CellTiter 96 AQ_ueous_ One Solution Cell Proliferation Assay (MTS) (Promega, Madison, WI) was added and incubated for 2 h at 37 °C after which absorption at 490 nm was determined by plate reader. The absolute GR_50_ was calculated using GraphPad Prism 9.0 software after adjusting for baseline growth rate (GraphPad Software, Boston, MA).

### RNA sequencing

For bulk sequencing, SCC-9 E and SCC-9 p-EMT cells were plated in triplicate at 250,000 cells/well in 6-well plates and incubated for 48 h before harvesting. All cells were collected using an RNeasy kit (Qiagen, Venlo, Netherlands). After DNase treatment, RNA quality was determined by a NanoDrop (Thermo Fisher Scientific, Inc., Waltham, MA) spectrophotometer and Qubit fluorometer (Invitrogen). Gene expression profiling using RNA-seq was performed by the Genomics Division of the Iowa Institute of Human Genetics using manufacturer recommended protocols for the Illumina Stranded mRNA Kit (Illumina). Briefly, 500 ng of DNase I-treated total RNA was used to prepare sequencing libraries using the Illumina stranded mRNA library preparation kit (Cat. #20040534, Illumina, Inc., San Diego, CA). The molar concentrations of the resulting indexed libraries were measured using the 2100 Agilent Bioanalyzer (Agilent Technologies, Santa Clara, CA) and combined equally into a pool for sequencing. The concentration of the library pool was measured using the Illumina Library Quantification Kit (KAPA Biosystems, Wilmington, MA) and sequenced on the Illumina NovaSeq6000 genome sequencer using 100 bp paired-end SBS chemistry.

For single-cell sequencing, approximately 10 000 SCC-9 P, SCC-9 E, and SCC-9 p-EMT cells were suspended in cold DPBS with 0.04% acetylated BSA. Viability was determined to be greater than 90% by Countess II Automated Cell Counter (Thermo Fisher Scientific, Inc., Waltham, MA). Sc-RNA library preparation, sequencing, and quantification was performed by Genomics Division of the Iowa Institute of Human Genetics. Barcoded samples were pooled and sequenced using an Illumina NovaSeq6000 at the Iowa Institute of Human Genetics.

### RT-qPCR

For baseline target expression, cells were plated on six-well plates below 80% confluency before being collected. For siRNA-influenced target expression, cells were plated in 24-well plates, treated with 20 pmol siRNA and Lipofectamine 2000 complexes, and collected after 48 h of knockdown. All cells were collected using an RNeasy kit (Qiagen, Venlo, Netherlands). RNA quality was determined by a NanoDrop (Thermo Fisher Scientific, Inc., Waltham, MA) spectrophotometer.

RNase-free water was used to dilute 1800 ng of RNA to a volume of 10 mL. To this, 1 mL of 10 mM dNTP Mix (Thermo Fisher Scientific, Inc., Waltham, MA) and 1 mL 50 mM Random Hexamer (Invitrogen, Waltham, MA) was added and heated at 65 °C for 5 min then placed on ice. To the RNA-Hexamer hybrids we added 4 mL 5× First Strand Buffer (Invitrogen, Waltham, MA), 2 mL 0.1 M DTT (Invitrogen, Waltham, MA) and 1 mL 40 U/mL RNase OUT (Thermo Fischer Scientific, Inc., Waltham, MA). The solution was heated at 25 °C for 2 min. To this, 1 mL 200 U/mL SuperScript II Reverse Transcriptase (Invitrogen, Waltham, MA) was added. The following parameters were used for cDNA synthesis: 25 °C for 10 min, 42 °C for 50 min, 75 °C for 15 min, and hold at 4 °C.

One microliter of cDNA was combined with 19 mL of Power SYBR Green PCR Master Mix containing 50 pmol of forward and reverse primers for each target (IDT, Coralville, IA). b-Actin was used as housekeeping control. The thermocycler parameters were: 95 °C for 10 min followed by 40 cycles of denaturation (95 °C for 15 s) and extension (60 °C for 1 min). Changes in expression were determined using the 2^-ddCT^ method. Relevant qPCR Primers can be found below.

**PCR Primers**

***ACTB*** **:**

5’-CAT GTA CGT TGC TAT CCA GGC-3’ (Forward)

5’-CTC CTT AAT GTC ACG CAC GAT-3’ (Reverse)

***EDIL3:***

5’-TGA GTG CCC AGG CGA ATT TAT-3’ (Forward)

5’-ACG TGC ATA GTA GGG ATA CCA TT-3’ (Reverse)

***NISCH:***

5’-AGT GTC CGC TTC TCA GCA AC-3’ (Forward)

5’-AGG CTC CCA CTC ATC AAA TTC T-3’ (Reverse)

***ITGA11*** **:**

5’-GTG GCA ATA AGT GGC TGG TC-3’ (Forward)

5’-GTT CCC GTG GAT CAC TGG AC-3’ (Reverse)

***VIM*** **:**

5’-GAC GCC ATC AAC ACC GAG TT-3’ (Forward)

5’-CTT TGT CGT TGG TTA GCT GGT-3’ (Reverse)

***ITGAV*** **:**

5’-ATC TGT GAG GTC GAA ACA GGA-3’ (Forward)

5’-TGG AGC ATA CTC AAC AGT CTT TG-3’ (Reverse)

***ZEB1*** **:**

5’-CAG CTT GAT ACC TGT GAA TGG G-3’ (Forward)

5’-TAT CTG TGG TCG TGT GGG ACT-3’ (Reverse)

***WNT5A*** **:**

5’-GCC AGT ATC AAT TCC GAC ATC G-3’ (Forward)

5’-TCA CCG CGT ATG TGA AGG C-3’ (Reverse)

***PTK2*** **:**

5’-GCT TAC CTT GAC CCC AAC TTG-3’ (Forward)

5’-ACG TTC CAT ACC AGT ACC CAG-3’ (Reverse)

**Negative siRNA Control:**

IDT Non-targeting Control (DS NC 1) (Product Number:51-01-14-04, Lot: 0000967011) (IDT, Coralville, IA).

***siEDIL3***:

rArGrArArGrCrArArArGrUrGrUrUrUrArCrUrArArUrGrACA (Sense)

rUrGrUrCrArUrUrArGrUrArArArCrArCrUrUrUrGrCrUrUrCrUrCrA (Antisense)

### ATAC-seq

Chromatin accessibility was profiled using the Active Motif ATAC-Seq Kit according to the manufacturer’s protocol. Briefly, 50 000 viable cells from each experimental group were collected, lysed, and incubated with Tn5 transposase to simultaneously fragment and tag accessible chromatin. Transposed DNA was purified, PCR-amplified, and assessed for quality and concentration. Libraries were sequenced on the Element Biosciences Aviti24 platform at Genomics Division of the Iowa Institute of Human Genetics using a Cloudbreak FS-High output kit and a 2 × 75 bp paired-end configuration.

Fastq paired-end reads were preprocessed using Cutadapt to remove leftover adapter sequences. Reads were then aligned to GRch38 using bwa [70]. Reads either belonging to the mitochondrial genome or reads marked as duplicates were filtered out. Broad and Narrow peaks were identified using MACS2 [71], and consensus peaks across samples were created and imported into a DESeq2 object, which was then loaded into the R statistical environment. Differential accessibility was assessed by comparing the called peaks across various conditions using DESeq [72]. Peak annotation was performed in R using the ChIPseeker package [73]. Pathway analysis for the KEGG, Reactome, and GO Terms databases was performed using the pathfindR [74] package.

Additionally, we performed gene set enrichment analysis using the Molecular Signatures Database (MSigDB) definitions [75], employing a hypergeometric test for each gene set. For multiple testing correction, we used the FDR method with a threshold of 0.05. Transcription Factor gene set enrichment was done using the ATACseqTFEA [76] Bioconductor package.

### CRISPR-KO invasion screen

SCC-9 p-EMT cells were transfected with lentivirus containing the lentiCas9-Blast plasmid from Addgene. LentiCas9-Blast was a gift from Feng Zhang (Addgene viral prep #52962-LV). Cas9-expressing cells were selected with 20 mg/mL blasticidin (Gibco, Waltham, MA), previously determined to kill all SCC-9 p-EMT cells after 48 h.

Cells were expanded to approximately 2 × 10^8^ cells across forty T175 flasks. Cells were divided into suspensions containing 6 × 10^6^ cells in 20 mL media with 10 mg/mL Polybrene (MilliporeSigma, Burlington, MA). To these suspensions, 1.8 × 10^6^ transduction units of lentiviral prep from Addgene of the Human CRISPR Knockout Pooled Library (Brunello) with lenti-Guide-Puro vector backbone were added. This library was a gift from David Root and John Doench (Addgene viral prep #73178-LV).

Cells were replated in T175 flasks for 48 h in 2 mg/mL puromycin (Gibco, Waltham, MA) for selection of sgRNA expression. Millicell 6-Well Hanging Inserts (8.0 mm PET) (MilliporeSigma, Burlington, MA) were coated with 400 mL 3.4 mg/mL PathClear Cultrex Basement Membrane Extract (Bio-Techne, Minneapolis, MN). sgRNA-expressing SCC-9 p-EMT cells were resuspended in 0% FBS media and seeded at 1 × 10^6^ cells/2 mL per insert. The bottom chamber contained 3 mL of 20% FBS media. Sixty replicates were used. Remaining cells were used to collect DNA using a QIAGEN DNeasy Blood & Tissue Kit (Qiagen, Venlo, Netherlands) for baseline library expression. Complete viral library RNA was collected using a QIAamp Viral RNA Mini Kit (Qiagen, Venlo, Netherlands) and converted to cDNA using the RT-qPCR protocol above.

After 48 h at 37 °C, cells embedded in the basement membrane extract were collected using Cell Recovery Solution (Corning, Corning, NY) and plated. Invading cells were collected by rinsing inserts with DPBS to remove FBS, followed by immersion in 1 mL of 0.25% trypsin for 2 min. Trypsin was inactivated with 100 mL FBS. This was repeated 6 times per insert. Invading cells were replated. Bottom chambers were rinsed with DPBS and covered with 10% FBS to preserve descendant cells. Cells recovered and expanded for 48 h. DNA from all control and experimental groups (viral library, baseline, non-invaders, invaders, and descendants) was collected using a QIAGEN DNeasy Blood & Tissue Kit (Qiagen, Venlo, Netherlands). DNA was amplified using nested PCR primers and barcoded using xGen UDI Primer Pairs (IDT, Coralville, IA). Sequencing was performed using an S2 flow cell with 150 bp paired-end reads. Nested PCR primer sequences are below.

**Nested PCR primers for screen library amplification**

**sgRNA**:

5’-CAAGTTGATAACGGACTAGCCTTATT-3’ (Forward)

5’-AATAAGGCTAGTCCGTTATCAACTTG-3’ (Reverse)

**P5-Left:** 5’-ACACTCTTTCCCTACACGACGCTCTTCCGATCTCCAATTCCCACTCCTTTCAAGACCT-3’

**P5N-Left:** 5’-ACACTCTTTCCCTACACGACGCTCTTCCGATCTNCCAATTCCCACTCCTTTCAAGACC-3’

**P5NN-Left:** 5’-ACACTCTTTCCCTACACGACGCTCTTCCGATCTNNCCAATTCCCACTCCTTTCAAGACC-3’

**P5-Right:** 5’-ACACTCTTTCCCTACACGACGCTCTTCCGATCTTTGTGGAAAGGACGAAACACCG-3’

**P5N-Right:** 5’-ACACTCTTTCCCTACACGACGCTCTTCCGATCTNTTGTGGAAAGGACGAAACACCG-3’

**P5NN-Right:** 5’-ACACTCTTTCCCTACACGACGCTCTTCCGATCTNNTTGTGGAAAGGACGAAACACCG-3’

**P7-Left:** 5’-GTGACTGGAGTTCAGACGTGTGCTCTTCCGATCTTTGTGGAAAGGACGAAACACCG-3’

**P7N-Left:** 5’-GTGACTGGAGTTCAGACGTGTGCTCTTCCGATCTNTTGTGGAAAGGACGAAACACCG-3’

**P7NN-Left:** 5’-GTGACTGGAGTTCAGACGTGTGCTCTTCCGATCTNNTTGTGGAAAGGACGAAACACCG-3’

**P7-Right:** 5’-GTGACTGGAGTTCAGACGTGTGCTCTTCCGATCTCCAATTCCCACTCCTTTCAAGACCT-3’

**P7N-Right:** 5’-GTGACTGGAGTTCAGACGTGTGCTCTTCCGATCTNCCAATTCCCACTCCTTTCAAGACCT-3’

**P7NN-Right:** 5’-GTGACTGGAGTTCAGACGTGTGCTCTTCCGATCTNNCCAATTCCCACTCCTTTCAAGACCT-3’

### Spatial transcriptomics

For our tissue sample, we chose an advanced-stage tongue cancer specimen with positive surgical margins following surgical resection. The patient was a 61-year-old male with a significant tobacco use history. Their final pathology demonstrated a 7.8 cm oral tongue, moderately differentiated, squamous cell carcinoma with lymphovascular invasion and bone invasion. The tumor was not cleared microscopically, and deep margins were positive in the oral cavity submucosa and pterygoid musculature. Neck dissection demonstrated 10/82 positive lymph nodes with extranodal extension, with final pathologic staging of pT4aN3b, and a clinical stage of IVB (American Joint Committee on Cancer 8th Edition). The sample was also notable because it aggressively recurred post-surgery, with PET-avid disease observed in the masticator space, cervical neck, and lungs within 4 weeks after surgery. The specimen was formalin-fixed and paraffin-embedded less than a year prior to our study. Histologic annotation was provided by an independent surgical pathologist to define regions.

After confirmation of adequate RNA quality (DV200 = 57%) within our tumor sample, the tissue was sectioned into four 5-micron-thick slices. The slices were processed using the Visium Spatial Gene Expression for Formalin-Fixed Paraffin-Embedded (FFPE) protocol (10x Genomics, Pleasanton, CA) by the NeuroBank Core of the University of Iowa. Tissue sections were mounted on a Visium Spatial Gene Expression slide. The FFPE tissue sections were then deparaffinized, stained, decrosslinked, and incubated with a proprietary RNA probe mixture. The tissue sections were stained with hematoxylin and eosin (H&E) to visualize histological features, which were annotated by an independent pathologist to designate regions of tumor, stroma, and other histological features. The tissue sections were hybridized to the probes and placed onto Visium Spatial Gene Expression slides containing spatially barcoded oligonucleotides. The sections were permeabilized to release the target RNA, which was captured by the spatial barcodes on the slides. Subsequent reverse transcription produced cDNA, preserving spatial information. The cDNA was then amplified to generate sequencing libraries, following the manufacturer’s protocols. These libraries were sequenced on an Illumina (San Diego, California, USA) platform to obtain high-resolution spatial gene expression data.

### ELISA

Cells were plated 200,000 cells/well in six-well plates and incubated for 24 h. Media was aspirated from plates and wells were washed with 36 °C 1× phosphate-buffered saline (PBS). F12/DMEM (Gibco^TM^; Thermo Fisher Scientific, Inc., Waltham, MA.) supplemented with 10% Fetal Bovine Serum (FBS; Corning®, Woodland, CA), 1% MEM Non-Essential Amino Acids (Gibco^TM^, Thermo Fisher Scientific, Inc., Waltham, MA.), and 1% Penicillin-Streptomycin-10 000 U/mL (Gibco^TM^, Thermo Fisher Scientific, Inc., Waltham, MA.) was added to the wells. Cells were incubated with this fresh media for 48 h. Conditioned media was then collected, spun at 500 g for 7 min at 4 °C, sterile filtered (0.2 mm), and frozen at -20°C until use. Six replicates per cell line were created from this protocol. Cells were also collected from each well and counted using 0.4% trypan blue (Invitrogen, Eugene, OR) and Invitrogen Countess II Automated Cell Counter (Invitrogen, Eugene, OR). One additional 6-well plate was created to act as negative control, no cells were added to this plate, but had media incubated/collected as described above. Samples were submitted to the University of Michigan Immune Monitoring Core for ELISA. All of the ELISA sets were measure using duoset kits from R&D Systems (Minneapolis, MN). Antibodies used include: EDIL3 (DY 6046-05), TGFb (DY 240), and MMP-2 (DY 902).

Data from the University of Michigan Immune Monitoring Core was received in pg/mL with each of the 6 replicates read twice and averaged. If sample data points were determined to be out of bounds of ELISA read, they were assigned the highest bound value (in pg/mL) for the specific target of the ELISA screen. Sample values were then subtracted from the negative control for each target. If this data resulted in a value between −2 and 0, they were assigned “0” for further analysis. Data was then normalized to the cell count collected in the ELISA Collection protocol.

### Bioinformatics

For bulk RNA sequencing analysis, paired-end reads were demultiplexed and converted to FASTQ format using Illumina’s ‘bcl2fastq’ utility and custom python scripts. FASTQ data were processed for RNA count quantification with nf-core/rnaseq (v3.6), a best-practices open-source workflow for RNA-seq data (https://nf-co.re, Nextflow version 22.04) [77–79]. Reads were aligned against the Ensembl reference GRCh38 using the STAR aligner [80]. Quality control was performed with qualimap and samtools, a computational tool that detects common QC problems [81–83]. Gene-level counts were used for differential gene expression analysis with DESeq2 [72]. mRNA differentially expressed pathway analysis was compared between SCC-9 EMT cell lines using iPathwayGuide software (Advaita Bioinformatics, Ann Arbor, MI) [84]. This software analysis tool implements the ‘Impact Analysis’ approach that takes into consideration the direction and type of all signals on a pathway.

For single-cell RNA sequencing, sequenced reads were processed using Nextflow (v24.04.4.5917) and the nf-core/scranseq (v4.0.0; https://doi.org/10.5281/zenodo.3568187) against the GRCh38 genome and the 10XV3 protocol parameters. We used Seurat (V5.10) [85] to perform quality control on the counts obtained from Nextflow. Each condition was loaded independently, and an integrated dataset was created by finding integration anchors. The combined dataset was scored for phase of cell cycle. During scaling, the cell cycle components were regressed out.

Pseudotime analysis was performed using monocle3 (v1.3.7) on the individual conditions [https://cole-trapnell-lab.github.io/monocle3/]. Time zero for each condition was selected manually in an epithelial-to-mesenchymal direction. Clustering was performed with a 1e^-1^ resolution. The pseudotime graph was learnt with “use.partition=FALSE”. All the plots were generated using ggplot2. All the analyses involving Seurat and monocle3 were performed inside the R statistical environment v4.4.1.

The sequenced reads from the CRISPR-KO Invasion Screen were processed using MAGECK v 0.5.9.5 [86]. In short, we used “mageck count” with the broadgpp-brunello-library-corrected sgRNA library [87] while also correcting for the non-targeting-control sequences. After counting, pairwise comparisons were performed using the “mageck test” command. To obtain the number of effective tests to find the *p*-value threshold for a given pairwise condition, the original count data was subsetted for the samples in those conditions. We obtained the Spearman rank correlation for the subsetted data and obtained the number of effective tests using the “meff” function of the poolR package (v1.1-1), with the “liji” method. The *p*-value threshold for significance is obtained by dividing 0.05 by the number of effective tests obtained by the liji method [88].

For the spatial transcriptomic data, Raw FASTQ files were processed using 10x Space-Ranger v2.0.1 with the Visium_Human_Transcriptome_Probe_Set_v1.0_GRCh38-2020-A transcriptome. The resulting quantified features and pathology slides were loaded into the R statistical environment (v3) using Seurat [89] (v4) through the Load10X_Spatial function. Seurat was used for all spatial transcriptomic analyses. The loaded data were normalized using SCTransform. To cluster the tissue spots, we performed PCA (RunPCA) and used the FindNeighbors Seurat function with 30 dimensions [90]. Then we found clusters (FindClusters) and performed UMAP clustering for visualization (RunUMAP). For each cluster, we found unbiased, only positive markers by running the FindAllMarkers function (only.pos = T). Correlation matrices between selected genes were produced by obtaining the Spearman rank correlation coefficients. Tissue plots and gene expression maps were generated using ggplot2.

Gene set enrichment was performed in R, using a one-sided Fisher exact (hypergeometric) test. Gene sets were obtained from the Molecular Signatures Database (MSigDB, https://www.gsea-msigdb.org/gsea/msigdb). Significant gene sets were defined as those with an FDR < 0.05. Immune cell composition was inferred using cell2location [91] implemented in Python. We used the provided “sp” reference dataset to train the model. We used the trained model on our Visium counts, using 30 as the estimated cell count per spot.

### Statistical analyses

All experiments were performed at least in triplicate to ensure scientific rigor and with negative and positive controls, where applicable. All statistical analyses were performed using GraphPad Prism 9.0 software (GraphPad Software, Boston, MA). Individual data points are shown with standard deviation. Statistical analyses were performed using T-tests to compare two means, one-way ANOVA for parametric data comparing more than two groups, and Kruskal-Wallis test for non-parametric data comparing more than two groups.

#### Ethics approval and consent to participate

All experiments and procedures described in this study were conducted in accordance with relevant institutional guidelines, regulations, and approved protocols. Human tissue studies were reviewed and approved by the University of Iowa Human Subjects Office Institutional Review Board (IRB ID #202207292). No live vertebrate animal studies were performed. Informed consent was obtained from the participant prior to collection and use of the tumor specimens for research purposes, including spatial transcriptomic analyses. Consent for publication was not required because this study does not contain any identifiable individual participant data or images.

## Supplementary information


Supplemental Figures 1-10
Updated Uncropped Immunoblots


## Data Availability

The datasets generated during and/or analyzed during the current study are available from the corresponding author on reasonable request.
